# WTAP-mediated m^6^A modification of FRZB triggers the inflammatory response via the Wnt signaling pathway in osteoarthritis

**DOI:** 10.1038/s12276-023-01135-5

**Published:** 2024-01-04

**Authors:** Xueying An, Rongliang Wang, Zhongyang Lv, Wenshu Wu, Ziying Sun, Rui Wu, Wenjin Yan, Qing Jiang, Xingquan Xu

**Affiliations:** 1 State Key Laboratory of Pharmaceutical Biotechnology, Division of Sports Medicine and Adult Reconstructive Surgery, Department of Orthopedic Surgery, Nanjing Drum Tower Hospital, The Affiliated Hospital of Nanjing University Medical School, 321 Zhongshan Road, Nanjing, 210008 Jiangsu P.R. China; 2 Branch of National Clinical Research Center for Orthopedics, Sports Medicine and Rehabilitation, Nanjing, P.R. China; 3 Department of Orthopedic, Affiliated Jinling Hospital, Medical School, Nanjing University, Nanjing, P.R. China

**Keywords:** Methylation, Osteoarthritis

## Abstract

Osteoarthritis (OA) is the most common form of arthritis. However, the exact pathogenesis remains unclear. Emerging evidence shows that N6-methyladenosine (m^6^A) modification may have an important role in OA pathogenesis. This study aimed to investigate the role of m^6^A writers and the underlying mechanisms in osteoarthritic cartilage. Among m^6^A methyltransferases, Wilms tumor 1-associated protein (WTAP) expression most significantly differed in clinical osteoarthritic cartilage. WTAP regulated extracellular matrix (ECM) degradation, inflammation and antioxidation in human chondrocytes. Mechanistically, the m^6^A modification and relative downstream targets in osteoarthritic cartilage were assessed by methylated RNA immunoprecipitation sequencing (MeRIP-seq) and RNA sequencing, which indicated that the expression of frizzled-related protein (FRZB), a secreted Wnt antagonist, was abnormally decreased and accompanied by high m^6^A modification in osteoarthritic cartilage. In vitro dysregulated WTAP had positive effects on β-catenin expression by targeting FRZB, which finally contributed to the cartilage injury phenotype in chondrocytes. Intra-articular injection of adeno-associated virus-WTAP alleviated OA progression in a mouse model, while this protective effect could be reversed by the application of a Wnt/β-catenin activator. In summary, this study revealed that WTAP-dependent RNA m^6^A modification contributed to Wnt/β-catenin pathway activation and OA progression through post-transcriptional regulation of FRZB mRNA, thus providing a potentially effective therapeutic strategy for OA treatment.

## WTAP: A Game-changer in Osteoarthritis Cartilage Degradation

This research examined the function of an RNA alteration known as WTAP in osteoarthritis (OA - a condition which breaks down the joints). Presently, treatments are limited due to insufficient understanding of the root causes of the disease. The study discovered that WTAP, an enzyme (a substance that speeds up chemical reactions in the body) that changes RNA, was increased in cartilage affected by OA and played a role in the advancement of the disease. This was accomplished through a process known as methylation, influencing the expression of FRZB, a protein that blocks a signaling pathway linked with cartilage growth and joint health. By inhibiting FRZB, WTAP activates this harmful pathway, resulting in cartilage breakdown. The study suggests that focusing on WTAP could be a potential treatment approach for OA.

This summary was initially drafted using artificial intelligence, then revised and fact-checked by the author.

## Introduction

Osteoarthritis (OA) is a degenerative disease that is significantly associated with disorders of cartilage metabolism, inflammation, and oxidative stress^1–3^. It has been estimated that by 2032, the prevalence of OA among individuals over 45 years old will rise to 29.5%^4^. However, the treatment of OA is still restricted due to an unclear understanding of its pathogenesis. Therefore, it is essential to investigate the pathogenic mechanisms of OA to develop effective therapies.

Recent advances in OA research have revealed that aberrant epigenetic alterations in OA-susceptible genes are linked to the development and progression of the disease, indicating that epigenetic mechanisms play a crucial role in the onset or progression of OA^5,6^. Common epigenetic modifications, including histone modifications^7–9^, DNA methylation^10–12^, and noncoding RNA regulation, have been extensively studied in OA^13,14^. In addition to reversible chemical modifications on DNA, similar modifications to RNA could be functional mediators of gene expression, and N6-methyladenosine (m^6^A) modification is one of the most deeply researched modifications^15^. The m^6^A modification of RNA is mainly driven by m^6^A methyltransferases (METTL3, METTL14, WTAP, and VIRMA, termed “writers”), removed by m6A demethylases (FTO and ALKBH5, termed “erasers”), and recognized by m^6^A binding proteins (IGF2BPs, YTHDCs, and YTHDFs, termed “readers”)^16^. Although this modification has been gradually discovered in various diseases and affects the development of diseases^17–19^, only a few studies have shown that m^6^A methylation in chondrocytes could affect OA progression, such as the methyltransferase METTL3^20–22^. Information on the roles of the remaining m^6^A methylation in OA is still lacking.

The wingless-type (Wnt) signaling pathway is essential for the development of cartilage, bone, and joints and has also been linked to postnatal joint homeostasis and disease^23^. The canonical Wnt signaling pathway, which involves β-catenin, has been extensively investigated in OA animal models as well as in OA patients^24^. Current data strongly suggest that canonical Wnt signaling is essential for joint and bone formation, as well as cartilage maintenance^25^. This phenotype is distinguished by protracted cell survival and the prevention of hypertrophic differentiation^26^.

Frizzled-related protein (FRZB) is an inhibitor of the Wnt pathway that restricts the activation of the canonical Wnt pathway^27^. The classic Wnt/β-catenin signaling pathway is abnormally activated in OA, and FRZB effectively alleviates the severity of OA by inhibiting the Wnt pathway^28,29^. In addition, the Wnt/β-catenin signaling pathway was reported to be regulated by m^6^A methylation and thus affected disease progression^30,31^. However, it remains unclear whether m^6^A methylation contributes to changes in FRZB expression and influences downstream Wnt pathway activation in OA.

In this study, we investigated the role of m^6^A writers and explored the underlying mechanisms in human osteoarthritic cartilage. Our results first revealed that WTAP expression was the most significantly upregulated methyltransferase in OA, and it promoted chondrocyte degeneration, inflammation and oxidative stress via its m^6^A catalytic activity. Importantly, we found that the decreased expression of WTAP inhibited Wnt signaling activation and alleviated OA progression by decreasing m^6^A-modified FRZB in vitro and in vivo.

## Materials and methods

### Clinical specimens

All human joint tissues were obtained from patients with detailed clinical characteristics from January 2020 to December 2021. The human samples and the experimental protocols were reviewed and approved by the Ethics Committee of the Nanjing Drum Tower Hospital (2020-156-01). Written informed consent was obtained from the patients before the start of the study.

### Animal study

Wild C57BL/6 mice were purchased from the Model Animal Research Center of Nanjing University and then kept at the institution’s animal facility with high standards of animal husbandry for subsequent experiments. Destabilization of the medial meniscus (DMM) surgery was performed on 12-week-old male mice under general anesthesia to generate the osteoarthritic phenotype. One week after the initial surgery, the mice were randomly treated with adeno-associated virus (AAV) negative control virus (NC) or siWTAP (General Biosystems, China), and then, the mice were treated with or without SKL2001. SKL2001 (6 mg/kg, every two days)^32^ and a total of 10 μl of solution containing AAV-siWTAP and NC (~1 × 10^11^ vg/ml) were slowly injected into the knees. At 8 weeks post-injury, the mice were euthanized, and the knee joints and major organs were collected. All animal feeding and experimental procedures were approved by the Ethics Committee and the Institutional Animal Care and Use Committee of Drum Tower Hospital, Nanjing University Medical School.

### Open field test

The wild-type C57BL/6 mice were divided into three groups: sham, DMM surgery with NC (DMM + NC), and DMM surgery with siWTAP (DMM+siWTAP). Then, each mouse was placed in a square open field, a camera system was used to monitor the activities of the animals for 5 min, and VisuTrack video was used to record and analyze the trajectory.

### Footprint analysis

Mice were painted with different ink colors on the paws of the forelimbs and hind limbs and then placed in a transparent tube with white paper on the bottom to record their tracks.

### Histological analysis

After fixation and decalcification, mouse knee joints and major organs were embedded in paraffin wax and sectioned into continuous thin slides (5 mm thick) by a microtome (Thermo, Germany). The bone sections were subjected to Safranin O/fast green (S.O.) staining (#G1371, Solarbio, Beijing, China) to evaluate the cartilage lesions using the Osteoarthritis Research Society International (OARSI) grading system. Organ sections were stained with hematoxylin and eosin (H&E) (#C0105S, Beyotime) for structural analysis.

### Immunohistochemical (IHC) staining

After deparaffinization and hydration, the histological slides of the mouse knee joint were subjected to antigen retrieval and blocked with 5% bovine serum albumin (BSA) for 1 h at room temperature. Following incubation overnight (4 °C) with primary antibodies against collagen II (#BA0533, Boster, Wuhan, China), ADAMTS5 (#14351, Cell Signaling Technology, USA), MMP13 (#ab219620, Abcam, UK), FRZB (#ab205284, Abcam, UK), and β-catenin (#8814S, Cell Signaling Technology), the slides were incubated with a horseradish peroxidase-conjugated secondary antibody (Biosharp, Shanghai, China) for 30 min at 37 °C. An ultrasensitive DAB Kit (Typing, Nanjing, China) was used to visualize the immunohistochemical staining.

### MeRIP-Seq and RNA-Seq analysis

Total RNA was extracted from uninjured and osteoarthritic cartilage tissues (2 females and 1 male with an average age of 69.67 ± 8.083 years), followed by mRNA sequencing and m^6^A sequencing, which were simultaneously performed (Genesky Biology, Shanghai, China). For mRNA sequencing, mRNAs were single-end sequenced with an Illumina HiSeq 2500. Transcript assembly and differential expression were evaluated by Cufflinks with Refseq mRNAs to guide fragmentation and were then incubated with m^6^A antibody (Synaptic System, Germa) for immunoprecipitation. Immunoprecipitated RNA was analyzed by high-throughput sequencing. Kyoto Encyclopedia of Genes and Genomes (KEGG) pathway and Gene Ontology (GO) analyses were employed to predict target gene functions.

### Cell culture and OA model in vitro

Human chondrocytes were isolated from human cartilage tissue (6 females and 7 males with an average age of 70.31 ± 5.663 years). Briefly, human cartilage pieces were cut into small pieces and digested with type II collagenase. Primary chondrocytes were cultured in DMEM (Gibco, CA, USA) containing 10% FBS (Gibco, CA, USA) and 1% penicillin/streptomycin (HyClone, USA) at 37 °C in a humidified atmosphere with 5% CO_2_. Experiments with chondrocytes were performed in passages 0–2. With recombinant human TNF-a (PeproTech, Rocky Hill, USA), osteoarthritis was induced in previously extracted human chondrocytes.

### Cell transfection

The WTAP interference virus (HBLV-h-WTAP shRNA1-ZsGreen-PURO) was purchased from Hanbio Biotech (Shanghai, China). Human chondrocytes were plated in a six-well plate and amplified to a cell density of 80%, after which a mixture of lentivirus (MOI = 100) and polybrene (6 µg/mL, Solarbio, Beijing, China) was added to the medium. After 48 h, the chondrocytes were screened with puromycin (Meilunbio, Dalian, China). For WTAP overexpression, 80% confluent chondrocytes were transfected with the plasmid pCMV-WTAP neomycin (GK Gene) and the FuGENE®HD transfection reagent (Promega, Madison, Wisconsin, USA) and incubated for 24 h at 37 °C and 5% CO_2_. Then, chondrocytes were screened using neomycin (Meilunbio, Dalian, China) for one day. All transfection efficiencies were verified by qRT‒PCR and Western blotting analysis.

### Immunofluorescence (IF)

Chondrocytes were seeded in 12-well plates at 1.5 × 10^5^ cells, after which the chondrocytes were washed with PBS and fixed with 4% paraformaldehyde for 15 min at room temperature. The cells were then blocked with 5% bovine serum albumin (BSA) for one hour at room temperature. The cells were incubated with the primary antibodies anti-WTAP (#60188-1-Ig, Proteintech, USA), anti-β-catenin (#8814S, Cell Signaling Technology, USA), and anti-FRZB (#ARG58723, Arigo, China) overnight at 4 °C, after which the chondrocytes were washed and incubated with Alexa Fluor 568-conjugated secondary antibody (Invitrogen, Carlsbad, USA) for 1 h in the dark. Finally, chondrocytes were incubated with a DAPI staining solution (AbMole, Houston, USA) for nuclear staining. Fluorescence was examined under a fluorescence microscope (Nikon, Tokyo, Japan).

### RNA isolation and quantitative RT‒PCR

Total RNA was isolated from chondrocytes using TRIzol (Invitrogen, Carlsbad, CA, USA) according to the manufacturer’s protocol. Complementary DNA (cDNA) was synthesized by Hiscript II QRT SuperMix for qPCR (+gDNA wiper) (Vazyme, Nanjing, China) with total RNA. Quantitative PCR was performed using ChamQTM SYBR Color qPCR Master Mix (Vazyme, Nanjing, China) with gene-specific primers. The primer sequences are shown in Supplementary Table 1. The β-actin gene was used as an internal control to normalize differences in each sample.

### Reactive oxygen species (ROS) staining

The total level of reactive oxygen species in the cell was detected with the H2DCFDA probe (AbMole, Houston, USA). According to the instructions, chondrocytes were seeded in 12-well plates at a 1.5 × 10^5^ cell/ml concentration, after which the adherent chondrocytes were incubated with 5 μM staining solution for 30 min at 37 °C in the dark. They were subsequently washed with PBS three times and immediately analyzed with oxidized H2DCFDA fluorescence under a fluorescence microscope (Nikon, Tokyo, Japan). Signal quantification was performed using ImageJ software (US National Institutes of Health).

### Western blotting

Cells were lysed in RIPA buffer with a protease inhibitor cocktail (KeyGEN, Jiangsu, China) to obtain protein lysates, which were then quantified with a BCA quantification kit (Beyotime, Shanghai, China). After SDS‒PAGE electrophoresis, the proteins were transferred to PVDF membranes (Millipore, Massachusetts, USA) and blocked in WB blocking solution at room temperature for 1 h (Thermo Fisher Scientific, USA), followed by incubation with specific antibodies such as anti-ADAMTS4 (#11865-1-AP, Proteintech, USA), anti-ADAMTS5 (#14351, Cell Signaling Technology, USA), anti-MMP13 (#ab219620, Abcam, UK), anti-WTAP (#60188-1-Ig, Proteintech, USA), anti-FRZB (#ab205284, Abcam, UK), anti-β-catenin (#8814S, Cell Signaling Technology, USA) and β-actin (#81115-1-RR, Proteintech, USA) at 4 °C overnight. After washing with 1% TBST for 30 min and incubation with secondary antibodies (1:5000, Abcam) for 2 h, the signal on the membranes was detected by a chemiluminescence system (Tanon 5200 Multi).

### m^6^A RNA methylation assay (colorimetric)

The m^6^A RNA Methylation Assay Kit (ab185912, Abcam, Shanghai, China) was used to measure the m^6^A level of mRNA. According to the instructions, 200 ng of mRNA was incubated with 80 μL of binding solution at 37 °C for 90 min, after which 50 μL of diluted capture antibody was added and incubated for 60 min at room temperature, and 50 μL of diluted detection antibody was added to each well for 30 min. After incubation with 100 μL of developing solution in the dark at room temperature for 10 min, stop solution was added to each sample, and the absorbance was measured at 450 nm.

### Annexin V/PI staining assay

Live-dead cell staining was identified by an Alexa Fluor® 488 annexin V/Dead Cell Apoptosis Kit (SAB, Maryland, USA) according to the manufacturer’s instructions. Briefly, staining solutions A and B were added to the prepared dyeing solution C at a ratio of 1:1000. After incubation at 4 °C in the dark for 15–20 min, the samples were washed with PBS three times. A fluorescence microscope (Nikon, Tokyo, Japan) was used to detect the results.

### Statistical analysis

The results are presented as the mean ± standard error of the mean (SEM). GraphPad Prism 6.02 (GraphPad Software, Inc., USA) was used for statistical analysis. Two-tailed Student’s t test or two-way ANOVA was conducted by Graph-Pad Prism Software. *P* values < 0.05, *P* values < 0.01 and *P* values < 0.001 indicated statistical significance (marked with *, **, ***, respectively).

## Results

### High expression of WTAP in clinical osteoarthritic cartilage and TNF-α-induced chondrocytes

**Fig. 1:**
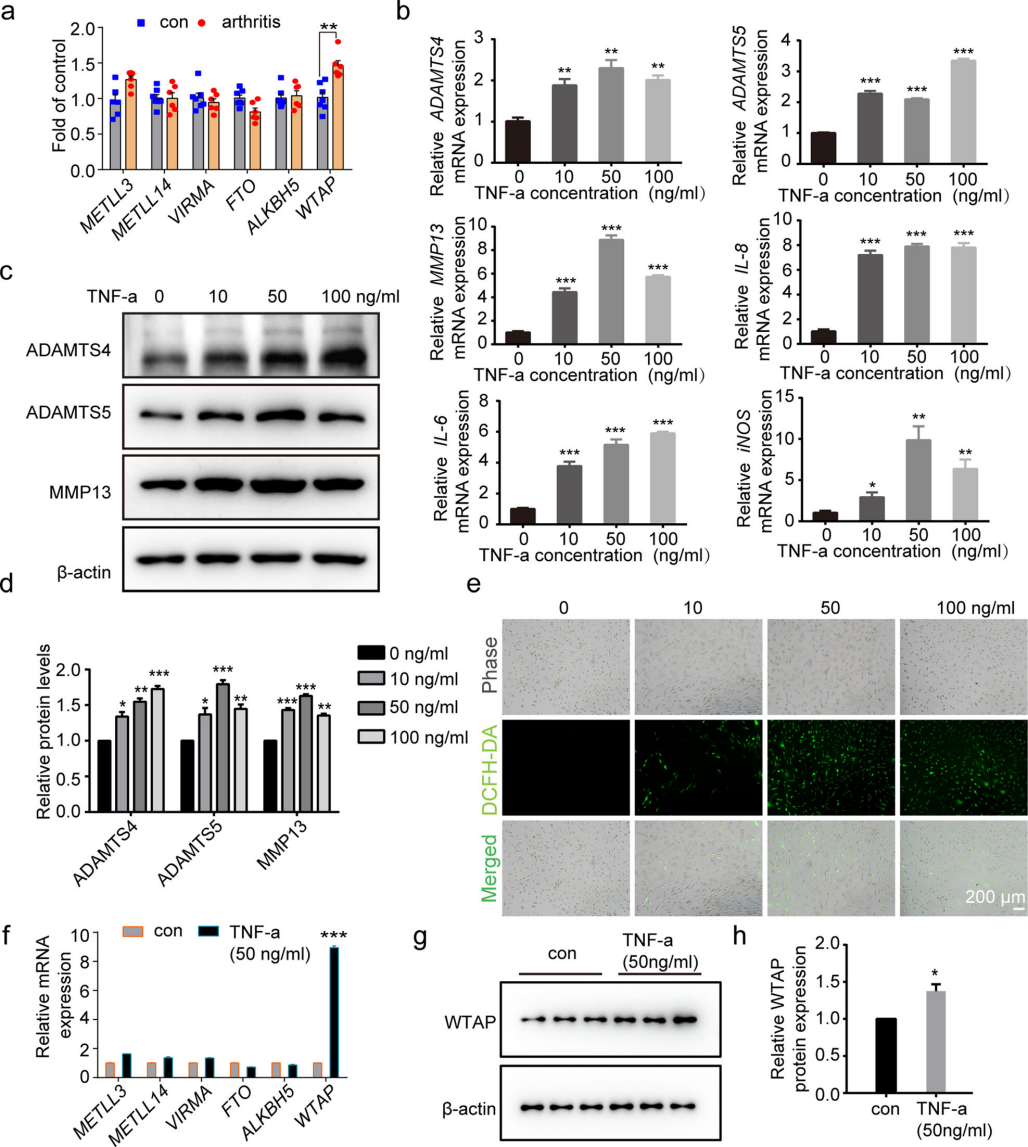
WTAP was upregulated in TNF-α-induced chondrocytes and osteoarthritic cartilage. **a** Relative mRNA expression of m^6^A methylases (*METLL3, METLL4, VIRMA*, *FTO*, *ALKBH5*, and *WTAP*) measured by qRT‒PCR in osteoarthritic (*n* = 6) vs. uninjured cartilage tissues (*n* = 7). **b** Relative mRNA expression of *ADAMTS4*, *ADAMTS5*, *MMP13*, *IL-6*, *IL-8*, and *iNOS* measured by qRT‒PCR in human chondrocytes with or without TNF-α treatment (50 ng/mL). **c**, **d** Western blotting (**c**) and quantitative analysis (**d**) of ADAMTS4, ADAMTS5, and MMP13 in human chondrocytes with or without TNF-α treatment (50 ng/mL). **e** DCFH-DA staining for ROS levels in chondrocytes treated with TNF-α (50 ng/mL). Scale bar: 200 µm. **f** Relative mRNA expression levels of m^6^A methylases (*METLL3*, *METLL4*, *VIRMA*, *FTO*, *ALKBH5*, and *WTAP*) measured by qRT‒PCR in chondrocytes with or without TNF-α treatment (50 ng/mL). **g**, **h** Western blotting (**g**) and corresponding quantitative analysis (**h**) of WTAP protein expression in osteoarthritic cartilage. Data are presented as the means ± SEMs. *P < 0.05; **P < 0.01; ***P < 0.001; ns, not significant.

First, we detected the expression of major m^6^A methylation-related genes (*METTL3*, *METTL14*, *WTAP*, *VIRMA*, *ALKBH5*, and *FTO*) in clinical osteoarthritic cartilage, and our results indicated that WTAP was the only methyltransferase with significantly upregulated expression (Fig. 1a). To induce a cellular OA model, we treated human chondrocytes with the proinflammatory cytokine TNF-a, a crucial inflammatory cytokine in OA^33,34^. Then, the OA-like phenotype was confirmed with increased mRNA expression of cartilage matrix metabolic genes (*ADAMTS4*, *ADAMTS5*, *MMP13*) and inflammatory genes (*IL-6*, *IL-8*, *iNOS*) (Fig. 1b) and elevated protein expression of ADAMTS4, ADAMTS5, and MMP13 (Fig. 1c, d). Since elevated levels of ROS in cartilage are associated with OA progression^4,35,36^, we found an increased ROS level in chondrocytes with TNF-α stimulation (Fig. 1e). Notably, chondrocytes treated with 50 ng/ml TNF-α for 24 h showed an obvious OA-like phenotype, which was applied in subsequent experiments.

Next, we investigated the expression of the major m^6^A methylation-related genes in the cellular OA model. In TNF-α (50 ng/ml)-induced chondrocytes, *WTAP* mRNA expression was the most significantly upregulated (Fig. 1f), and we also confirmed the upregulated WTAP protein expression in the chondrocytes treated with TNF-α (Fig. 1g, h). These results demonstrated that WTAP was highly expressed in osteoarthritic cartilage and may play a role in OA progression.

### Involvement of WTAP in the osteoarthritic chondrocyte phenotype

**Fig. 2:**
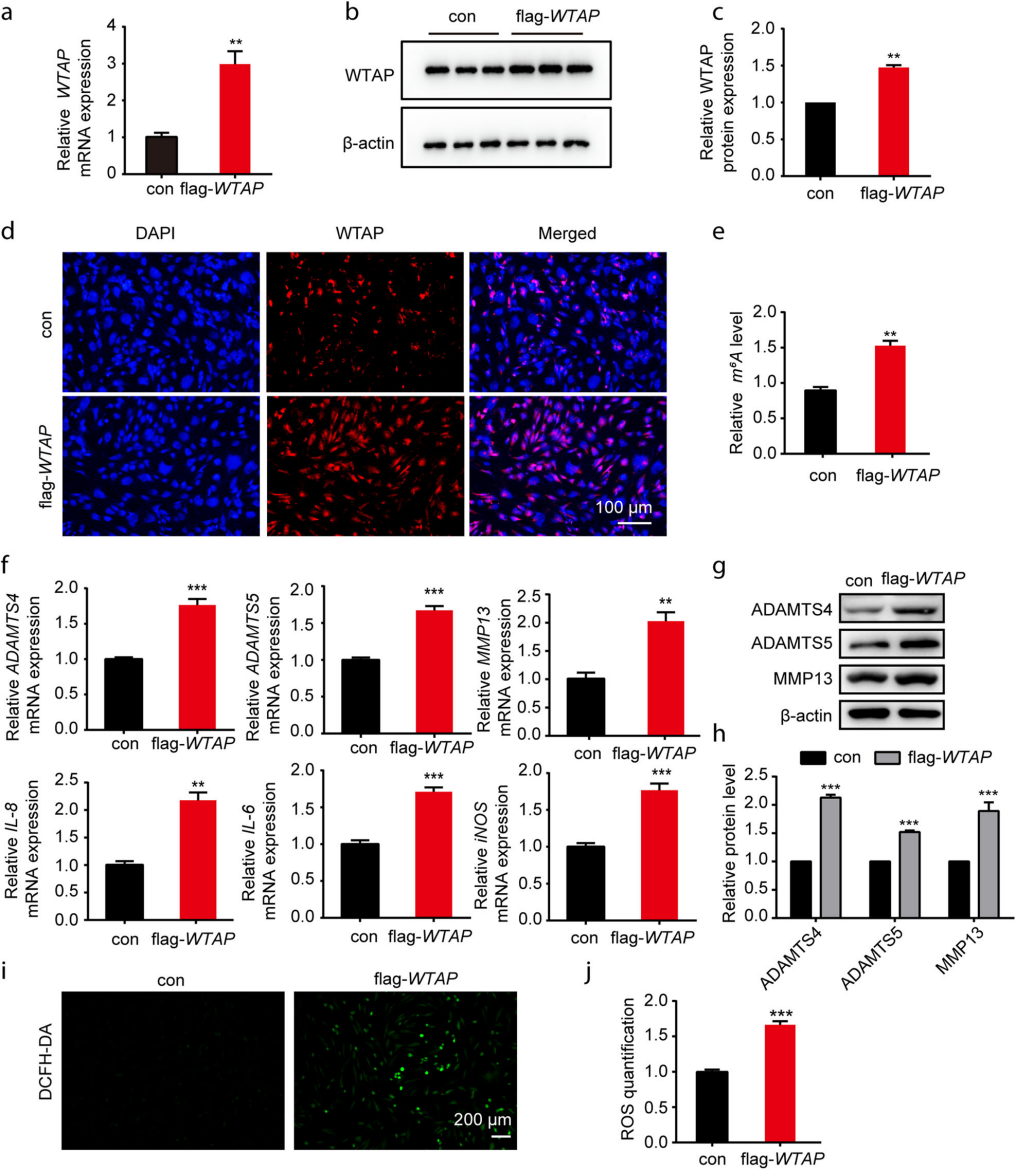
WTAP overexpression aggravated the TNF-α-induced osteoarthritic chondrocyte phenotype. **a**–**d** Overexpression efficiency of WTAP in human chondrocytes. Relative *WTAP* expression measured by qRT‒PCR (**a**), Western blotting (**b**), quantitative analysis (**c**), and IF staining (**d**) in chondrocytes after transfection with flag-*WTAP*. Scale bars: 100 µm. **e** The m^6^A level of total RNA in chondrocytes with or without *WTAP* overexpression. **f** Relative mRNA expression of *ADAMTS4*, *ADAMTS5*, *MMP13*, *IL-6*, *IL-8*, and *iNOS* was measured by qRT‒PCR in chondrocytes with or without WTAP overexpression. **g**, **h** Western blotting (**g**) and quantitative analysis (**h**) of ADAMTS4, ADAMTS5, and MMP13 expression in chondrocytes with or without *WTAP* overexpression. **i**, **j** Detection of ROS (**i**) and quantitative analysis (**j**) in chondrocytes with or without *WTAP* overexpression. Scale bars: 200 µm. Data are presented as the means ± SEMs. *P < 0.05; **P < 0.01; ***P < 0.001; ns, not significant.

To determine the role of WTAP in OA, we transfected chondrocytes with a plasmid overexpressing *WTAP* (flag-*WTAP*). After neomycin screening (10 mg/mL) for 24 h (Supplementary Fig. 1a), increased *WTAP* mRNA and protein levels were confirmed by qRT‒PCR and Western blotting (Fig. 2a–c). Immunofluorescence detection results also showed that the WTAP protein level was increased in chondrocytes (Fig. 2d), and the m^6^A methylation level was elevated following WTAP overexpression (Fig. 2e).

To evaluate the effect of WTAP on the osteoarthritic chondrocyte phenotype, we determined the mRNA expression levels of *ADAMTS4, ADAMTS5, MMP13, IL-6, IL-8*, and *iNOS* (Fig. 2f), along with the protein expression levels of ADAMTS4, ADAMTS5 and MMP13, and found that they were significantly increased in chondrocytes after overexpressing WTAP (Fig. 2g, h). Moreover, the production of active oxygen in the *WTAP*-overexpressing chondrocytes was significantly increased compared to that in the control chondrocytes (Fig. 2i, j).

**Fig. 3:**
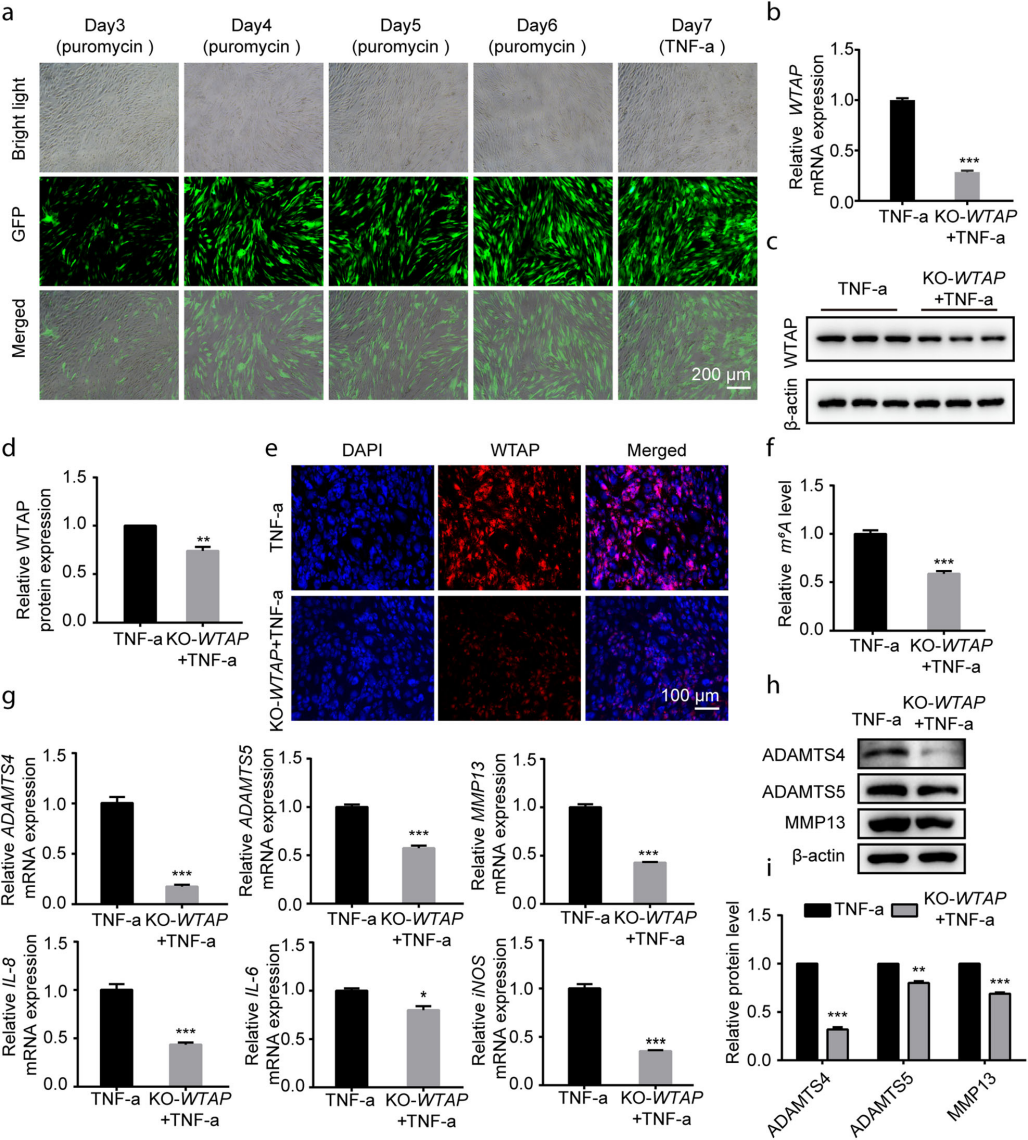
Knocking out WTAP alleviated the osteoarthritic chondrocyte phenotype. **a** GFP identification in human chondrocytes transfected with WTAP gene knockout lentivirus (KO-*WTAP*). Scale bars: 200 µm. **b**–**e** Knockout efficiency of WTAP in human chondrocytes. Relative *WTAP* expression measured by qRT‒PCR (**b**), Western blotting (**c**), quantitative analysis (**d**), and IF staining (**e**) of chondrocytes after transfection with KO-*WTAP*. **f** The m^6^A level of total RNA in chondrocytes with or without KO-*WTAP* transfection. **g** Relative mRNA expression of *ADAMTS4*, *ADAMTS5*, *MMP13*, *IL-6*, *IL-8,* and *iNOS* measured by qRT‒PCR in chondrocytes with or without KO-*WTAP* transfection. **h**, **i** Western blotting (**h**) and quantitative analysis (**i**) of ADAMTS4, ADAMTS5 and MMP13.

Furthermore, chondrocytes were transfected with lentivirus to knock out *WTAP* (KO-*WTAP*), and the optimal puromycin concentration was 2.5 μg/mL after screening the chondrocytes (Supplementary Fig. 1b). TNF-α (50 ng/mL) treatment had no effect on transfection efficiency on the seventh day (Fig. 3a). The KO-*WTAP* cell model demonstrated significantly reduced levels of *WTAP* mRNA and protein under TNF-α treatment (Fig. 3b–d). Immunofluorescence results also confirmed the effective transfection of WTAP lentivirus (Fig. 3e). Knocking out WTAP in chondrocytes decreased the m^6^A level (Fig. 3f) and significantly decreased the mRNA (*ADAMTS4, ADAMTS5, MMP13, IL-6, IL-8, iNOS*) and protein levels of arthritis-related genes (ADAMTS4, ADAMTS5, MMP13) in the OA cell model (Fig. 3g–i). Additionally, TNF-α-induced ROS levels were reduced in the chondrocytes with WTAP knockout (Supplementary Fig. 2a, b). Taken together, these results demonstrated the involvement of WTAP in the osteoarthritic chondrocyte phenotype.

### Enhanced m^6^A modification of FRZB in osteoarthritic cartilage

**Fig. 4:**
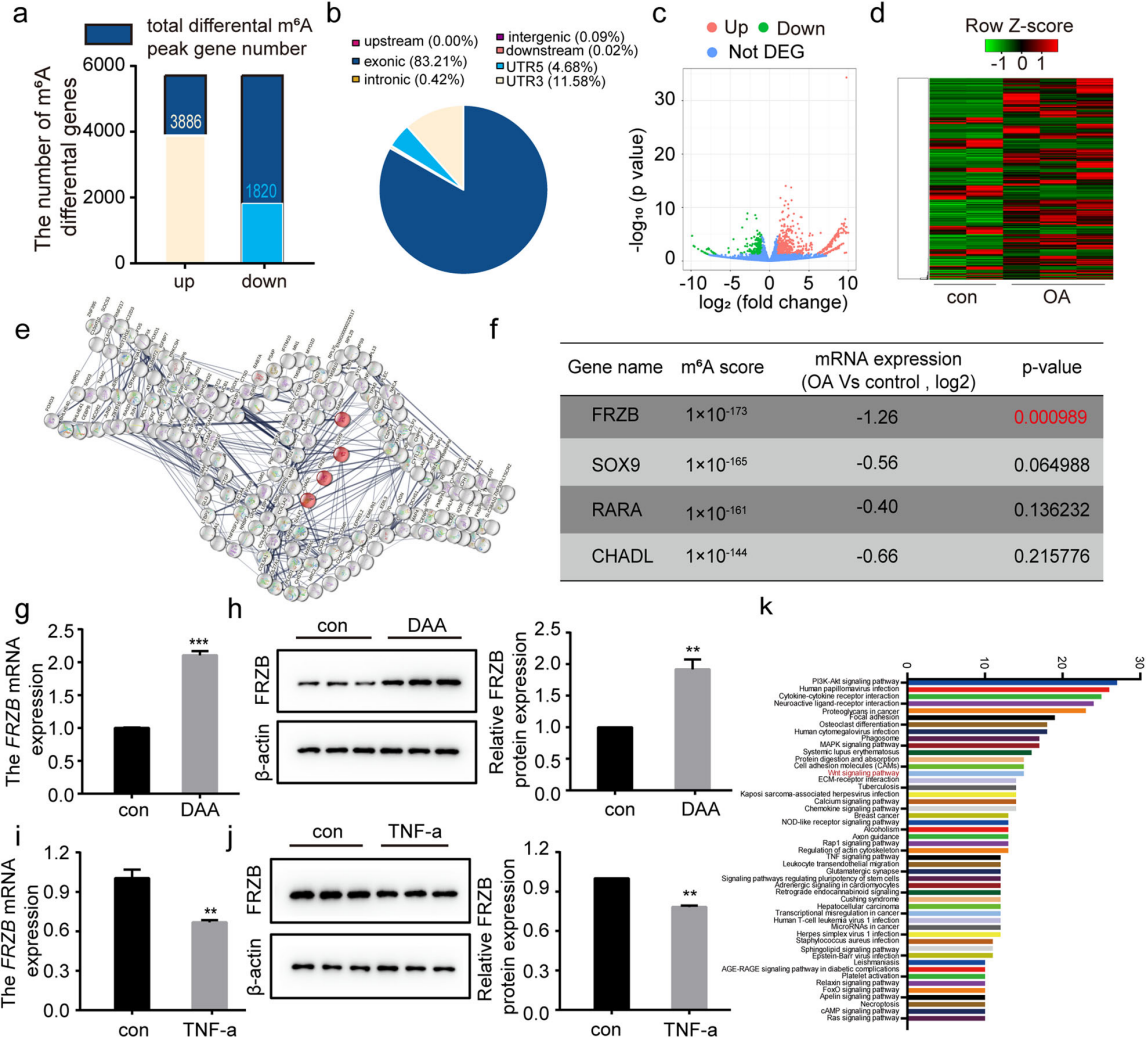
The Wnt pathway inhibitor FRZB abnormally elevated m^6^A methylation in OA. **a** Statistical analysis of m^6^A peak gene number. **b** The distribution of different m^6^A peaks in different regions of the reference genome. **c** Volcano plot illustrating the distributions of differentially expressed m^6^A genes. **d** Heatmap of differential gene clustering. **e** Functional analysis of hypermethylated genes based on the STRING database (the genes marked in red are genes related to cartilage development, https://cn.string-db.org/). **f** Cartilage development-related genes corresponding to the methylation sequencing score, gene expression and *P* value score. **g**, **h** FRZB expression was assessed by qRT‒PCR (**g**) and Western blotting (**h**) in human chondrocytes with or without treatment with the m^6^A methylation inhibitor DAA (50 µM). **i**, **j** FRZB expression was assessed in human chondrocytes treated with TNF-α (50 ng/ml). Relative *FRZB* expression measured by qRT‒PCR (**i**), Western blotting and quantitative analysis (**j**) of FRZB protein in human osteoarthritic chondrocytes. **k** KEGG analysis of m^6^A difference genes in osteoarthritic (*n* = 3) vs. normal cartilage tissues (*n* = 2). Data are presented as the means ± SEMs. *P < 0.05; **P < 0.01; ***P < 0.001; ns, not significant.

MeRIP-seq was performed in this study to determine the potential downstream targets of m^6^A modification in clinical osteoarthritic cartilage samples. Compared to uninjured cartilage, we identified 5706 genes that were modified by m^6^A in osteoarthritic cartilage (Fig. 4a). Among these genes, 3886 were found to have elevated methylation peaks, which were mainly enriched in the exon region (83.21%), followed by the 3’ UTR (11.58%) (Fig. 4b). Moreover, RNA-seq was performed in human osteoarthritic cartilage samples and uninjured cartilage, and there were 675 upregulated genes and 260 downregulated genes in osteoarthritic cartilage (Fig. 4c, d). To identify enriched functional pathways, we performed statistical analysis based on the top 200 upregulated genes according to the m^6^A score using the STRING database for protein‒protein interaction network functional enrichment analysis (http://string-db.org) (Fig. 4e). The m^6^A-modified genes associated with osteoarthritic cartilage were enriched in GO:0061037 (negative regulation of cartilage development) according to GO analysis. FRZB had the most significantly downregulated expression with identified high m^6^A modification in osteoarthritic cartilage (Fig. 4f).

To confirm the expression and modification of FRZB in OA, we examined its mRNA and protein levels in chondrocytes treated with DAA (50 µM), a specific m^6^A methylation inhibitor (Supplementary Fig. 3a). As shown in Fig. 4g, h, FRZB expression was found to be upregulated in response to DAA treatment, and we observed decreased FRZB expression in the cellular OA model (Fig. 4i, j). All of the above results were consistent with the results of our MeRIP-seq and RNA-seq analyses.

FRZB, a frizzled-related protein, is a Wnt antagonist that has been implicated in the regulation of Wnt/β-catenin signaling^37^. The results of Kyoto Encyclopedia of Genes and Genomes (KEGG) enrichment analysis also showed that the FRZB-related Wnt/β-catenin pathway was significantly enriched in clinical osteoarthritic cartilage (Fig. 4k). Hence, these findings suggest that WTAP may mediate the m^6^A modification of FRZB cells and regulate the osteoarthritic chondrocyte phenotype via the Wnt/β-catenin pathway.

### WTAP activated the Wnt/β-catenin signaling pathway via FRZB

**Fig. 5:**
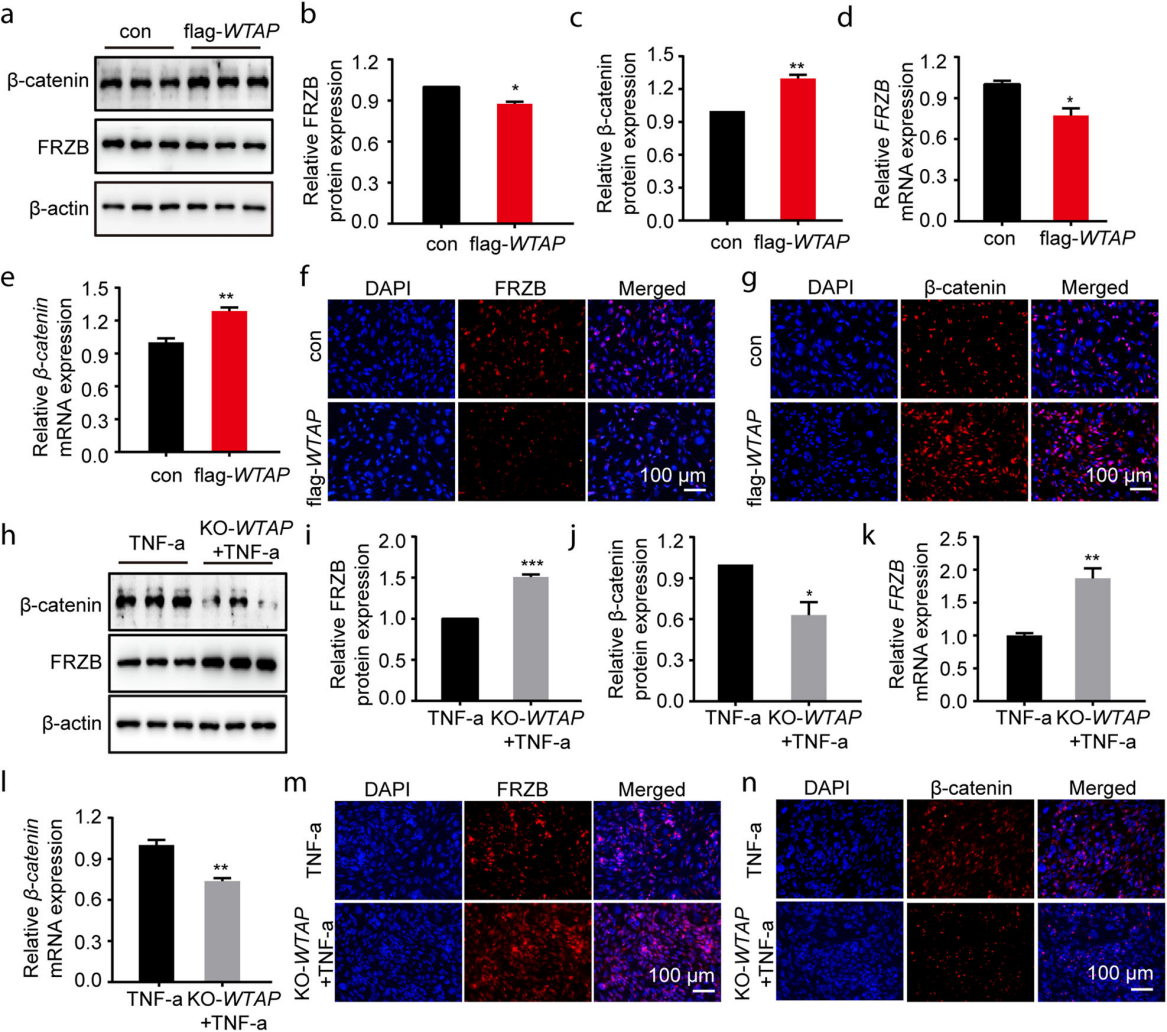
The FRZB-Wnt/β-catenin axis was regulated by WTAP in chondrocytes. **a**–**c** Protein levels of FRZB and β-catenin were analyzed in chondrocytes with or without *WTAP* overexpression. **d**, **e** Relative mRNA expression levels of *FRZB* (**d**) and *β-catenin* (**e**) were measured by qRT‒PCR in chondrocytes with or without *WTAP* overexpression. **f**, **g** IF staining of intracellular FRZB (**f**) and β-catenin (**g**) in TNF-α-pretreated cells with or without *WTAP* overexpression. Scale bars: 100 µm. **h**–**j** Protein levels of FRZB and β-catenin were analyzed in TNF-α-pretreated chondrocytes with or without KO-*WTAP* transfection. **k**, **l** Relative mRNA expression levels of *FRZB* (**k**) and *β-catenin* (**l**) were measured by qRT‒PCR in TNF-α-pretreated cells with or without KO-*WTAP* transfection. **m**, **n** IF staining of intracellular FRZB (**m**) and β-catenin (**n**) expression in TNF-α-pretreated cells with or without KO-*WTAP* transfection. Scale bars: 100 µm. Data are presented as the means ± SEMs. *P < 0.05; **P < 0.01; ***P < 0.001; ns, not significant.

To investigate whether WTAP affected the Wnt/β-catenin pathway, we identified the expression of FRZB and β-catenin in chondrocytes with WTAP overexpression (flag-*WTAP*) and WTAP knockout (KO-*WTAP*). In flag-*WTAP* chondrocytes, we observed a decrease in FRZB expression and an increase in β-catenin expression (Fig. 5a–e). Similar results were also confirmed by immunofluorescence (Fig. 5f, g). In contrast, we found increased FRZB expression and decreased β-catenin expression in the KO-*WTAP* chondrocytes treated with TNF-a (50 ng/ml) (Fig. 5h–l), which was also confirmed by immunofluorescence results (Fig. 5m, n). Hence, our results suggested that WTAP could activate the Wnt/β-catenin pathway by inhibiting FRZB expression.

### WTAP-induced the osteoarthritic chondrocyte phenotype through the m^6^A-FRZB/Wnt/β-catenin signaling pathway

**Fig. 6:**
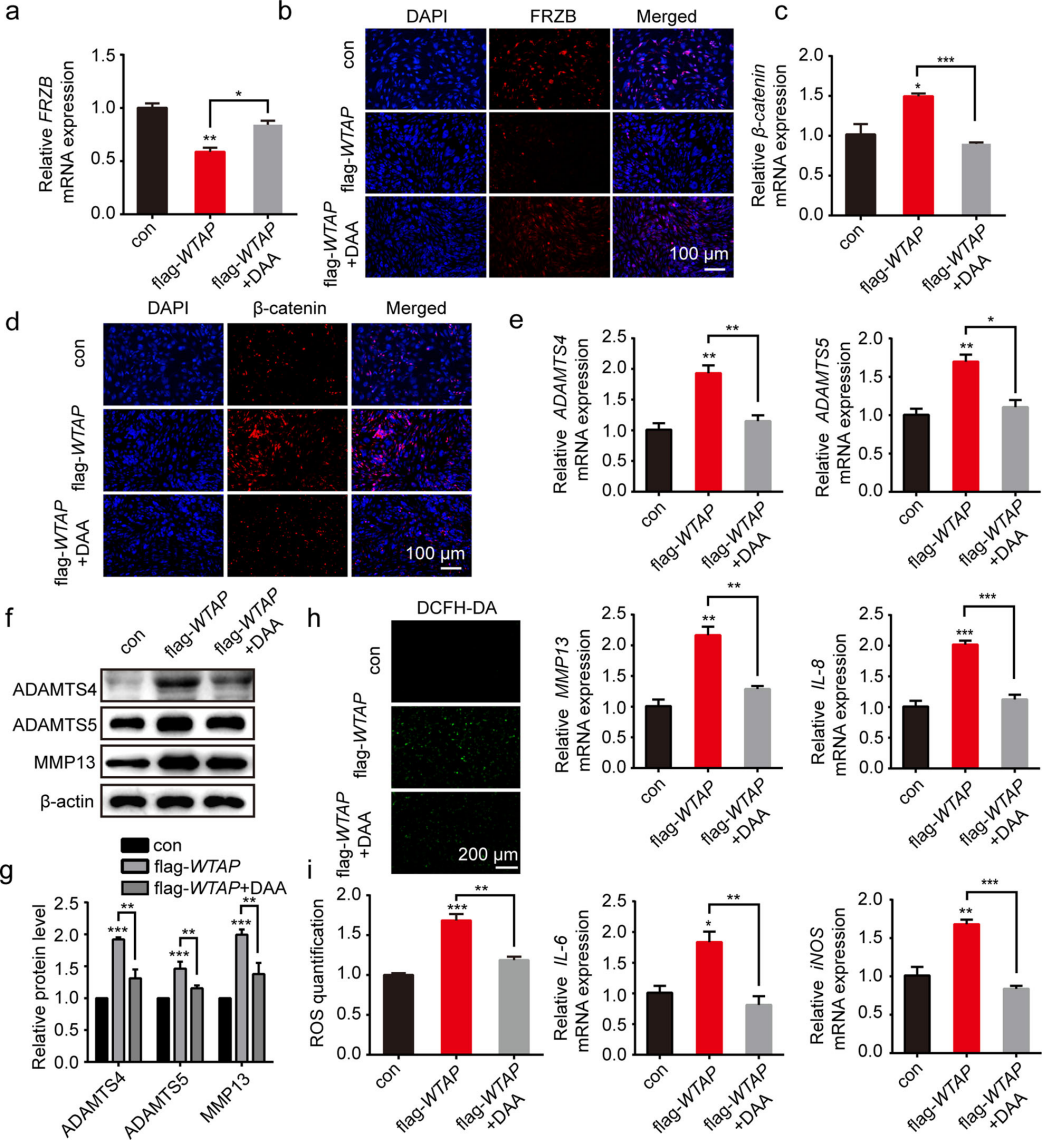
WTAP regulated Wnt/β-catenin signaling activation and the osteoarthritic chondrocyte phenotype by m^6^A modification of FRZB. **a**, **b** FRZB expression was analyzed in chondrocytes with *WTAP* overexpression after DAA (50 µM) treatment by qRT‒PCR (**a**) and IF staining (**b**). Scale bars: 100 µm. **c**, **d** β-catenin expression was analyzed in chondrocytes with *WTAP* overexpression after DAA (50 µM) treatment by qRT‒PCR (**c**) and IF staining (**d**). Scale bars: 100 µm. **e** Relative mRNA expression of *ADAMTS4*, *ADAMTS5*, *MMP13*, *IL-6*, *IL-8*, and *iNOS* measured by qRT‒PCR in chondrocytes with *WTAP* overexpression after DAA (50 µM) treatment. **f**, **g** Western blotting (**f**) and quantitative analysis (**g**) of ADAMTS4, ADAMTS5 and MMP13 expression in chondrocytes with *WTAP* overexpression after DAA (50 µM) treatment. **h**, **i** Detection of ROS (**h**) and quantitative analysis (**i**) in chondrocytes with *WTAP* overexpression after DAA (50 µM) treatment. Scale bars: 200 µm. Data are presented as the means ± SEMs. *P < 0.05; **P < 0.01; ***P < 0.001; ns, not significant.

To further determine WTAP-mediated m^6^A-FRZB in osteoarthritic cartilage, we used the methylation inhibitor DAA to investigate FRZB m^6^A modification and the activation of Wnt/β-catenin in the flag-*WTAP* chondrocytes. The qRT‒PCR and Western blotting results indicated that the mRNA and protein expression of FRZB was significantly upregulated in chondrocytes after treatment with DAA (50 µM) (Fig. 6a, b), while the mRNA and protein levels of Wnt/β-catenin were suppressed (Fig. 6c, d). Furthermore, we observed a significant decrease in the osteoarthritic chondrocyte phenotype in the *flag-WTAP* chondrocytes after DAA treatment, including decreased mRNA levels of arthritis-related genes (*ADAMTS4, ADAMTS5, MMP13, IL-6, IL-8, iNOS)* and decreased protein levels of ADAMTS4, ADAMTS5, and MMP13 (Fig. 6e–g). ROS staining showed that the level of reactive oxygen species was reduced in the *flag-WTAP* chondrocytes treated with DAA (Fig. 6h, i). Taken together, our findings suggested that WTAP affected the stability of FRZB by m^6^A modification to promote osteoarthritic chondrocyte catabolism.

### Silencing WTAP alleviated OA development through the Wnt/β-catenin signaling pathway

**Fig. 7:**
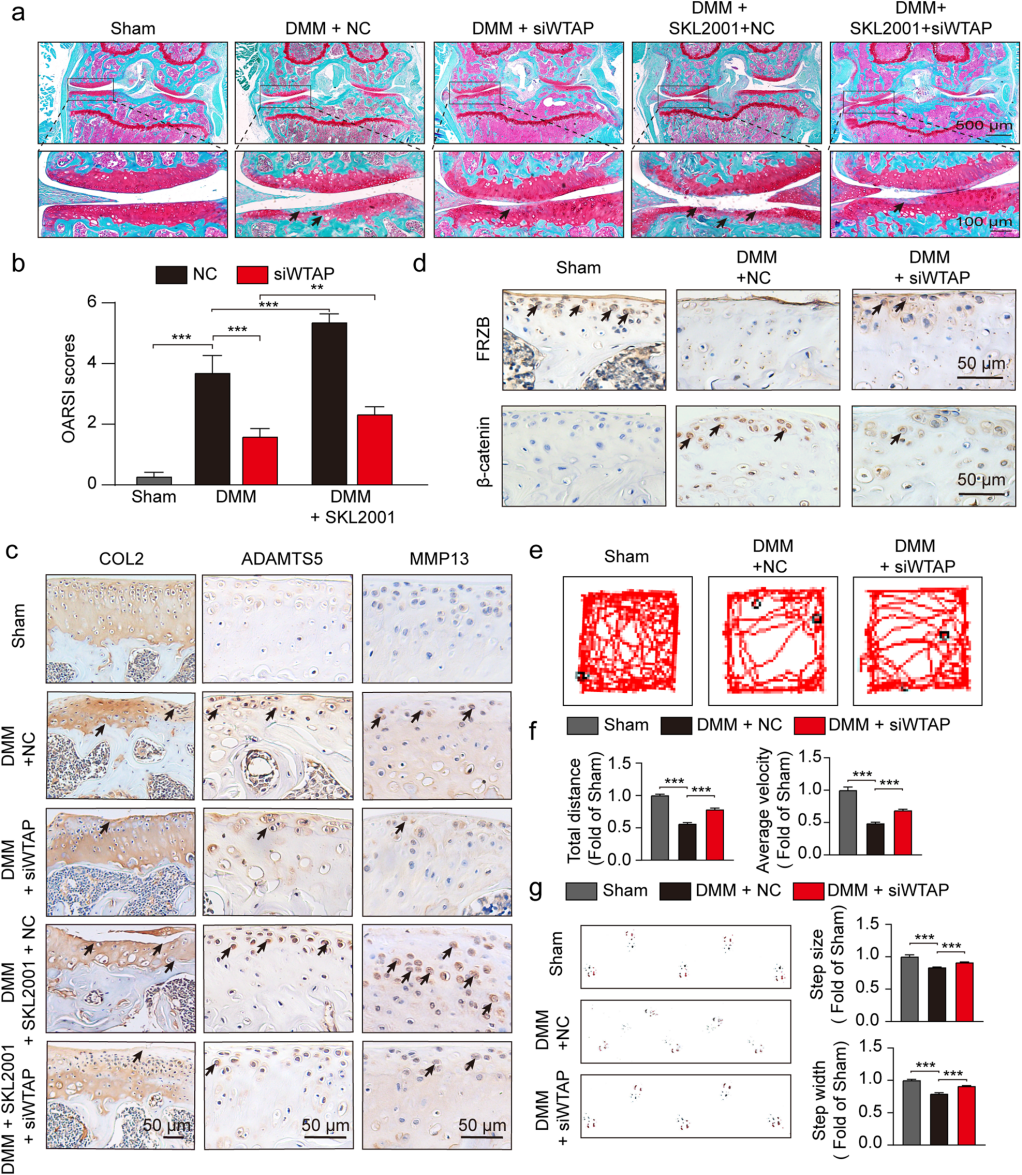
WTAP inhibition alleviated OA progression through Wnt/β-catenin signaling inactivation. **a** Safranin O/fast green staining of the cartilage in the indicated groups at 8 weeks after DMM surgery. **b** OARSI scoring of knee OA was evaluated by Safranin O/fast green staining (*n* = 6). **c**, **d** IHC staining of COL2, ADAMTS5, MMP13, FRZB, and β-catenin in cartilage from the indicated groups at 8 weeks after DMM surgery. **e** Open field test in different groups. **f** The corresponding quantitative analysis of (**e**) (*n* = 6). **g** Footprint images in different groups and statistical analysis (*n* = 6). Data are presented as the means ± SEMs. *P < 0.05; **P < 0.01; ***P < 0.001; ns, not significant.

To investigate whether WTAP plays a role in OA progression in vivo, we intra-articularly administered NC or adeno-associated virus (AAV) siWTAP to a DMM-induced OA mouse model. The knockout efficiency was confirmed by IHC (Supplementary Fig. 4a). Safranin O and fast green staining showed an improvement in cartilage surfaces of the mice with DMM-induced OA treated with AAV siWTAP but not NC AAV (Fig. 7a). Quantitative analysis with OARSI scoring showed that NC AAV led to significantly higher OARSI scores, whereas siWTAP treatment decreased OARSI scores (Fig. 7b). The injection of AAV siWTAP alleviated degenerative changes in the cartilage matrix, such as catabolic response and increased extracellular matrix (ECM) composition, in the OA mouse model, as indicated by the IHC results (Fig. 7c). Consistent with the in vitro findings, siWTAP deactivated the Wnt/catenin-β pathway, leading to improved osteoarthritic cartilage degradation by inhibiting the expression of FRZB (Fig. 7d). We also noted that these protective effects of siWTAP on osteoarthritic cartilage and OARSI scores were abolished through activation of the Wnt/β-catenin signaling pathway by SKL2001 (Fig. 7a–d). Moreover, we confirmed that siWTAP inhibits osteoarthritic phenotypes through the open field test and footprint analysis (Fig. 7e–g). Notably, the good biocompatibility of siWTAP and SKL2001 was observed in the tissue organizational structure in major organs (Supplementary Fig. 5a). These results demonstrate that the β-catenin pathway contributes to WTAP-stimulated catabolism changes in osteoarthritic cartilage and development.

## Discussion

**Fig. 8:**
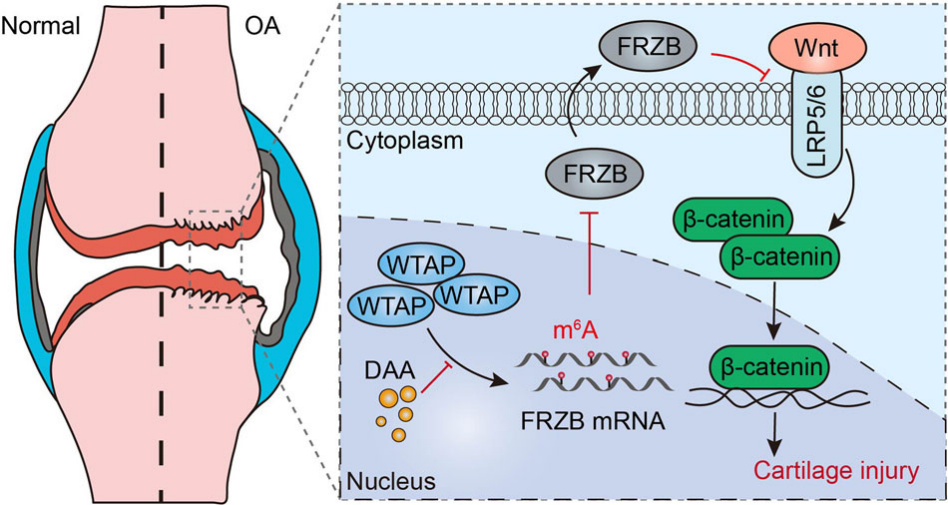
Proposed model for the regulation of WTAP on OA progression through FRZB/β-catenin signaling activation.

In the present study, we found that the m^6^A ‘writer’ WTAP had the most significant differential expression in osteoarthritic cartilage. MeRIP-seq and RNA-seq analysis revealed that possible targets of WTAP were associated with FRZB and the Wnt/β-catenin signaling pathway. The negative regulator of WTAP on FRZB expression and Wnt/β-catenin pathway inhibition was mediated by m^6^A modification, which finally induced degradation of the extracellular matrix, inflammatory reactions, and oxidative stress in osteoarthritic chondrocytes. Our study revealed a novel role of WTAP in OA progression and identified it as a promising therapeutic target for OA treatment (Fig. 8).

Among the numerous post-transcriptional modifications, m^6^A methylation, the most abundant mRNA modification, has a critical role in various physiological processes and disease progression^38^. In particular, m^6^A modification promotes the inflammatory response, chondrocyte apoptosis, and extracellular matrix imbalance in OA, which ultimately leads to OA exacerbation^39^. M^6^A-related enzymes determine the function and importance of m^6^A. For instance, m^6^A writers (METTL3, METTL14, WTAP, and VIRMA) interact with various binding proteins to regulate the methylation of RNA. In contrast, m^6^A erasers (FTO and ALKBH5) can catalyze RNA demethylation. Additionally, m^6^A readers (IGF2BPs, YTHDCs, and YTHDFs) recognize m^6^A-mediated physiological behavior and influence RNA function. Although much research has focused on m^6^A writers^40^ or erasers^16^ in osteoarthritic chondrocytes, there is a lack of studies on the general profile of m^6^A-related enzyme expression. Our study demonstrated that WTAP, the only notable methyltransferase, was responsible for osteoarthritic chondrocytes in OA patient samples and in vitro OA cellular models. Moreover, selective silencing of WTAP in articular cartilage effectively attenuated surgically induced cartilage damage and OA progression. These results indicate that the emergence of m^6^A regulation provides new insights into the molecular mechanisms of OA.

Furthermore, we first confirmed the possible targets of m^6^A modification in osteoarthritic cartilage in the present study. Our bioinformatic analysis revealed that FRZB had extensive m^6^A sites in osteoarthritic cartilage compared to the m^6^A sites of transcribed genes in uninjured cartilage based on m^6^A sequencing analysis. FRZB, also known as Sfrp-3, is a Wnt signaling pathway antagonist that belongs to the family of secreted frizzled-related proteins (sFRPs). Recent genetic findings suggested that FRZB could affect the cartilage metabolic matrix and cartilage degeneration^41,42^ and could even be defined as a potential therapeutic target for OA. Hence, it is crucial to understand the molecular basis of m^6^A regulation of FRZB expression in articular chondrocytes. Furthermore, we found that WTAP could affect the expression stability of FRZB by m^6^A modification, leading to cartilage degeneration and OA progression. These findings suggested that WTAP-mediated hyperm^6^A methylation inhibited FRZB expression in chondrocytes, which contributed to OA pathogenesis.

Wnt/β-catenin, as a downstream pathway of FRZB, has been implicated in the maintenance of cartilage homeostasis in OA, such as cartilage matrix catabolism, oxidative stress, and chondrocyte differentiation^43,44^. Previous studies have shown that m^6^A methylation-related enzymes, including m^6^A writers and erasers, regulate proteins in the Wnt/β-catenin signaling pathway, which affects disease progression^31,45,46^. However, to the best of our knowledge, no relative study on OA has confirmed the role of m^6^A regulation in Wnt/β-catenin signaling activation. Our study revealed that the genes with the most m^6^A modification differences, e.g., FRZB, according to m^6^A sequencing and transcription sequencing in osteoarthritic cartilage, were mostly involved in Wnt/β-catenin signaling. Furthermore, the activation of β-catenin by SKL2001 markedly reversed the protective effect of siWTAP on OA progression, suggesting that WTAP promotes cartilage matrix catabolism through Wnt/β-catenin signaling activation. Our results indicated that WTAP-mediated hyperm^6^A-methylated conditions were closely associated with Wnt/β-catenin pathway activation in osteoarthritic cartilage, highlighting a promising therapeutic target for OA treatment in the future.

The present study has some limitations. First, the influence of WTAP-induced m^6^A modification on the FRZB/Wnt/β-catenin axis was not further explored in chondrocytes under TNF-α exposure, which may cause some bias in OA conditions in vitro. Second, the FRZB/Wnt/β-catenin axis is not the only pathway that WTAP regulates, so the detailed regulation of WTAP in biological processes in OA needs further validation. Finally, our findings are based solely on laboratory data, and therefore, prospective clinical studies are needed to confirm the protective effect of WTAP inhibition in human OA progression.

In conclusion, our study revealed that the elevation of WTAP-dependent RNA m^6^A modification aggravated osteoarthritic cartilage degeneration and progression. Mechanistically, WTAP stabilized *FRZB* mRNA in an m^6^A-dependent manner and activated the Wnt/β-catenin signaling pathway in osteoarthritic chondrocytes. Our findings indicated that dysregulated RNA m^6^A modification mediated by WTAP may be a promising therapeutic target for OA treatment.

### Supplementary information

Supplementary material

## Data Availability

All data relevant to the study are included in the article or uploaded as supplementary information.
