# Supplementary material for: WTAP-mediated m6A modification of FRZB triggers the inflammatory response via the Wnt signaling pathway in osteoarthritis
Source: Exp Mol Med. 2024 Jan 4;56(1):156–67. doi: 10.1038/s12276-023-01135-5 (PMC10834961; doi:10.1038/s12276-023-01135-5)
Supplement: Supplementary file 1 — Supplementary material [file 12276_2023_1135_MOESM1_ESM.pdf]

WTAP-mediated m⁶A modification of FRZB triggers the inflammatory response via the Wnt signaling pathway in osteoarthritis

Xueying An[1,2,4], Rongliang Wang[1,2,4], Zhongyang Lv[3], Wenshu Wu[1,2], Ziying Sun[3], Rui Wu[1], Wenjin Yan[1,2*], Qing Jiang [1,2*], Xingquan Xu[1,2,5*]

[1] State Key Laboratory of Pharmaceutical Biotechnology, Division of Sports Medicine and Adult Reconstructive Surgery, Department of Orthopedic Surgery, Nanjing Drum Tower Hospital, The Affiliated Hospital of Nanjing University Medical School, 321 Zhongshan Road, Nanjing 210008, Jiangsu, P.R. China.

[2] Branch of National Clinical Research Center for Orthopedics, Sports Medicine and Rehabilitation, P.R. China.

[3] Department of Orthopedic, Affiliated Jinling Hospital, Medical School, Nanjing University.

[4] These authors contributed equally.

[5] Lead Contact

*Correspondence: xuxingquan@nju.edu.cn (X. Xu), qingj@nju.edu.cn (Q. Jiang), 13814533004@163.com (W. Yan).

Figure Legend

Supplementary Fig. 1 a. Cell viability assay of human chondrocytes with different concentrations of neomycin treatment at 24 h (n=7). b. Cell viability assay of human chondrocytes with different concentrations of puromycin treatment at 24 h (n=8).

Data are presented as means ± SEM. *P < 0.05; **P < 0.01; ***P < 0.001; ns, not significant.

Supplementary Fig. 2 a. Detection of ROS in TNF-a-pretreated chondrocytes with or without KO-*WTAP* treatment. Scale bars: 200 µm. b. The quantitative analysis of (a) (n=3).

Data are presented as means ± SEM. *P < 0.05; **P < 0.01; ***P < 0.001; ns, not significant.

Supplementary Fig. 3 a. The cell viability of human chondrocytes with different concentrations of 3-deazaadesine (DAA, n=8) treatment.

Data are presented as means ± SEM. *P < 0.05; **P < 0.01; ***P < 0.001; ns, not significant.

Supplementary Fig. 4 a. IHC staining of WTAP in different groups.

Supplementary Fig. 5 a. H&E-stained images of major organs of mice in different groups.

Supplementary Table 1 primer sequence.

Supplementary Fig. 1

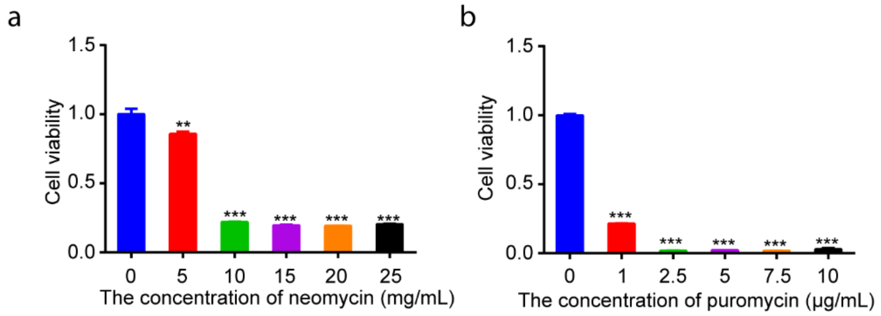

Supplementary Fig. 2

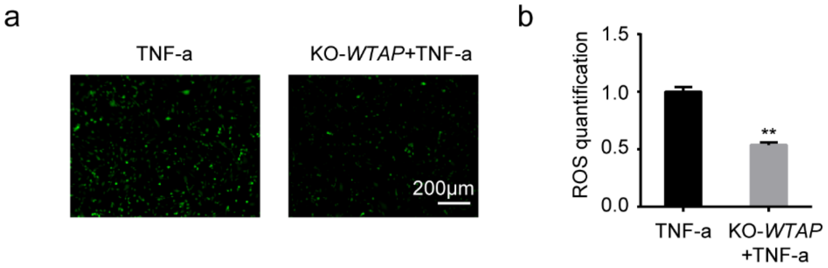

Supplementary Fig. 3

a

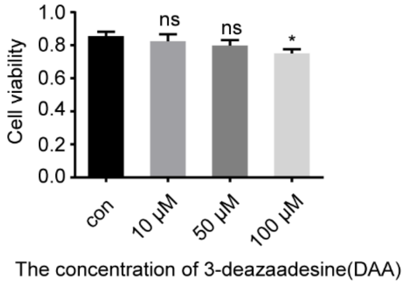

Supplementary Fig. 4

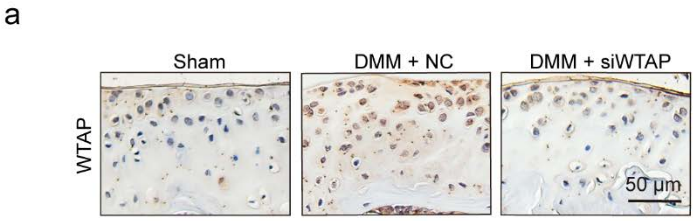

Supplementary Fig. 5

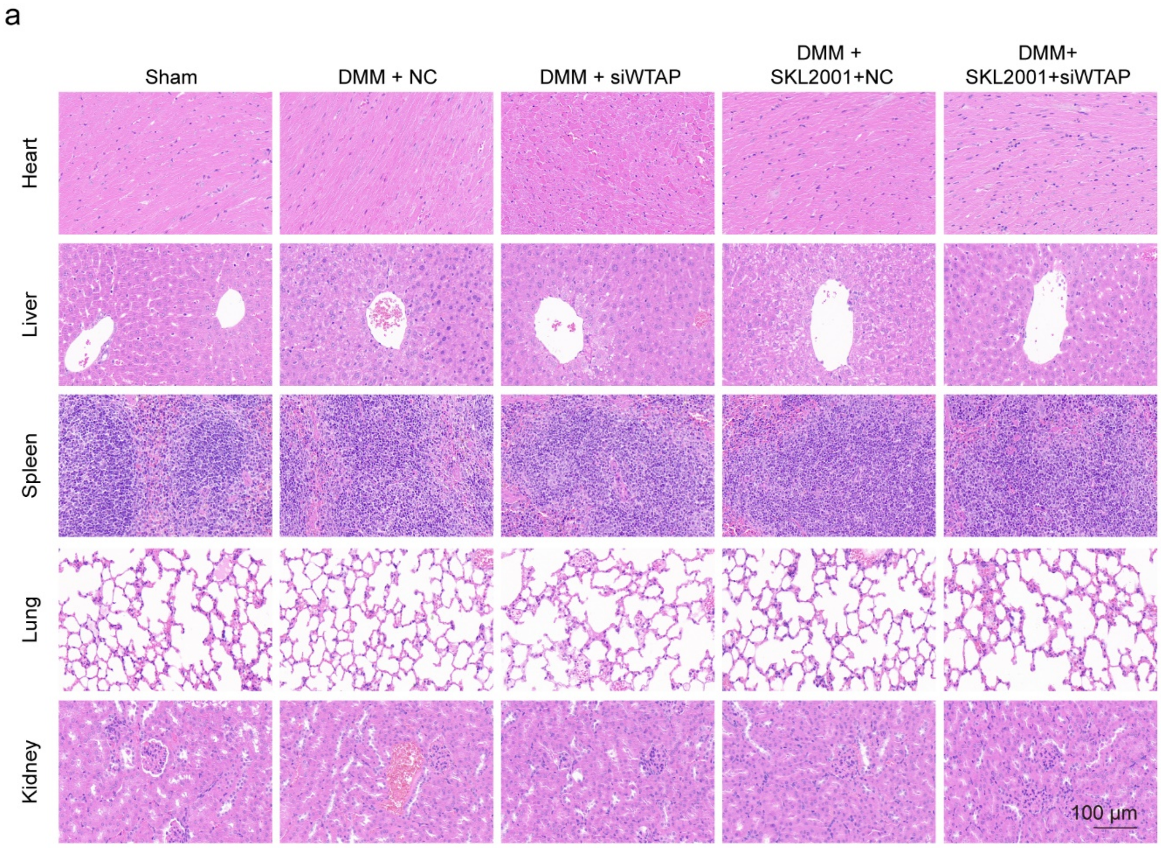

Supplementary Table 1. primer sequence.

Gene name	forward 5'-3'	reverse 5'-3'
ADAMTS4	TCACTGACTTCCTGGACAATGGC	GGTCAGCATCATAGTCCTTGCC
ADAMTS5	TTGGCCTCTCCCATGACGAT	CGTGGTAGGTCCAGCAAACA
MMP13	CGGCCATCAAGGGAAGAACTA	GAACCCTCTTTGGGTGAGGAA
IL8	ATGCTTCCCCTTAGCATTTTGT	CCAACATTCTGTGGAGGACACT
IL6	AGACAGCCACTCACCTCTTCAG	TTCTGCCAGTGCCTCTTTGCTG
iNOS	GCTCTACACCTCCAATGTGACC	CTGCCGAGATTTGAGCCTCATG
FRZB	GCTACACAGAAGACCTATTTCCG	CCGTGGAATGTTTACCAGAGAGG
β-catenin	CACAAGCAGAGTGCTGAAGGTG	GATTCCTGAGAGTCCAAAGACAG
β-actin	CACCATTGGCAATGAGCGGTTC	AGGTCTTTGCGGATGTCCACGT
